# Selective Expression of Flt3 within the Mouse Hematopoietic Stem Cell Compartment

**DOI:** 10.3390/ijms18051037

**Published:** 2017-05-12

**Authors:** Ciaran James Mooney, Alan Cunningham, Panagiotis Tsapogas, Kai-Michael Toellner, Geoffrey Brown

**Affiliations:** 1 Institute of Immunology and Immunotherapy, College of Medical and Dental Sciences, University of Birmingham, Edgbaston, Birmingham B15 2TT, UK; c.mooney@smd15.qmul.ac.uk (C.J.M.); alan.cunningham43@gmail.com (A.C.); k.m.toellner@bham.ac.uk (K.-M.T.); 2 Developmental and Molecular Immunology, Department of Biomedicine, University of Basel, Basel 4058, Switzerland; panagiotis.tsapogas@unibas.ch; 3 Institute of Clinical Sciences, College of Medical and Dental Sciences, University of Birmingham, Edgbaston, Birmingham B15 2TT, UK

**Keywords:** Flt3, growth factor receptors, cytokines, HSC, hematopoiesis, leukemia

## Abstract

The fms-like tyrosine kinase 3 (Flt3) is a cell surface receptor that is expressed by various hematopoietic progenitor cells (HPC) and Flt3-activating mutations are commonly present in acute myeloid and lymphoid leukemias. These findings underscore the importance of Flt3 to steady-state and malignant hematopoiesis. In this study, the expression of Flt3 protein and *Flt3* mRNA by single cells within the hematopoietic stem cell (HSC) and HPC bone marrow compartments of C57/BL6 mice was investigated using flow cytometry and the quantitative reverse transcription polymerase chain reaction. Flt3 was heterogeneously expressed by almost all of the populations studied, including long-term reconstituting HSC and short-term reconstituting HSC. The erythropoietin receptor (EpoR) and macrophage colony-stimulating factor receptor (M-CSFR) were also found to be heterogeneously expressed within the multipotent cell compartments. Co-expression of the mRNAs encoding Flt3 and EpoR rarely occurred within these compartments. Expression of both Flt3 and M-CSFR protein at the surface of single cells was more commonly observed. These results emphasize the heterogeneous nature of HSC and HPC and the new sub-populations identified are important to understanding the origin and heterogeneity of the acute myeloid leukemias.

## 1. Introduction

The hematopoietic stem and progenitor cells (HSPC) that give rise to all of the blood and immune cell types are within a small fraction of bone marrow cells that lack lineage markers (Lin^−^) and express Sca-1 and c-kit (LSK). HSPC are heterogeneous and multi-color flow cytometry has greatly aided the sub-fractionation of the LSK compartment, for example, on the basis of CD34 expression [1]. However, how to best phenotypically delineate populations of HSPC with differing biological properties is, as yet, uncertain.

A marker that is used to sub-divide LSK is the cell surface receptor fms-like tyrosine kinase 3 (Flt3/Flk2) which is a class III tyrosine kinase with structural homology to the c-kit and macrophage colony-stimulating factor (M-CSF/CSF1) receptors [2,3]. Flt3 was first identified on murine hematopoietic progenitor cells (HPC) [3] while expression in humans is restricted to CD34^+^ bone marrow cells, which include hematopoietic stem cells (HSC) [4]. Within the LSK compartment, only the Flt3^−^ fraction is capable of long-term myeloid reconstitution which has led to the viewpoint that Flt3 expression is linked to a loss of self-renewal capacity [5,6,7]. The promoter region of the Flt3 gene (*Flt3*) in Flt3^−^ LSK cells is occupied in a primed state [8] and the use of Flt3-Cre:loxp-eYFP mice has revealed that *Flt3* expression occurs within a phenotypically defined HSC compartment [9]. However, when LSK eYFP^+^ and eYFP^−^ cells from Flt3-Cre: loxp-eYFP mice are transplanted into secondary recipients only the latter provide robust myeloid reconstitution [9]. Boyer and colleagues have confirmed that all hematopoietic cells develop from HSC via a Flt3^+^ progenitor [10]. Together, the above results provide strong evidence to support the viewpoint that Flt3 protein can be first detected at the multipotent progenitor (MPP) stage during murine hematopoiesis. However, Flt3 may be expressed at a low level during earlier developmental stages and it remains unknown whether such expression might mark functionally distinct HSPC.

Dimerization of Flt3 occurs upon binding of its ligand (Flt3L) resulting in auto-phosphorylation of tyrosine residues [11,12], recruitment of the adapter proteins SHC, CBL and GRB [13,14,15] and signaling via the phosphoinositide 3 kinase (PI3K) and RAS pathways [16,17]. PI3K signaling is important to cell survival and, accordingly, the ligand promotes the survival and growth of hematopoietic progenitors, particularly myeloid and B lymphoid pathway progenitors [18,19,20]. The use of semi-solid medium assays has revealed that Flt3L influences the formation of granulocyte-macrophage (GM) colonies by human bone marrow CD34^+^ cells [21]. Flt3L also synergizes with other cytokines. The addition of Flt3L to interleukin (IL)-3 or IL-6 doubles the cell number in the colonies derived from mouse Lin^−^ Thy^lo^ Sca-1^+^ bone marrow cells and FltL combined with IL-3 or granulocyte-macrophage colony-stimulating factor (GM-CSF) enhances the growth of Lin^−^ CD34^+^ CD33^+^ human fetal liver progenitor cells [22]. Flt3L alone has little or no effect on these populations [19,23,24,25,26]. Flt3L has also been shown to synergize with the GM-CSF-IL-3 fusion protein Pixy 321 for human HPC [21] and with stem cell factor, GM-CSF, IL-6, IL-7, IL-11 and IL-12 for both murine and human HPC [23,24,25,26,27,28,29,30]. Importantly, Flt3L alone or combined with other appropriate cytokines does not affect the growth of the erythroid (BFU-E and CFU-E) [23,26,28] or megakaryocyte colonies in vitro [25,31,32]. In essence, the range of action of Flt3 is restricted to cells belonging to the lymphoid and GM pathways.

Flt3L^−/−^ mice have a reduced bone marrow, spleen and lymph node cellularity, and decreased numbers of dendritic cells (DC), Gr-1^+^ CD11b^+^ myeloid cells and lymphoid cells, including innate lymphoid cells [33,34]. Injection of Flt3L into mice leads to leukocytosis which is mostly due to an elevation in monocytes. The absolute number of LSK in bone marrow, spleen and peripheral blood is increased, lymphocytes are elevated, and there is a significant decrease in the hematocrit value and a 90% reduction in immature TER119^+^ erythroid cells [35]. Ceredig and colleagues injected mice with Flt3L and observed a 50% expansion of Flt3^+^ CD19^−^ B220^+^ CD117^lo^ cells, termed Early Progenitors with Lymphoid and Myeloid potential, and an increase in the number of DC [36,37]. Similarly, transgenic mice that express supra-physiological levels of human Flt3L (Flt3L-Tg) have increased numbers of Gr-1^+^ CD11b^+^ myeloid cells, NK1.1^+^ cells and DC. Studies of Flt3L-Tg mice have led to the proposition that Flt3L above a certain threshold level instructs myeloid and lymphoid development at the expense of cells developing along the megakaryocytic and erythroid (MegE) pathways, as these mice are anemic, thrombocytopenic and have a 9.7-fold decrease in megakaryocyte-erythrocyte progenitors (MEP) [38].

Blast cells of most cases of acute myeloid leukemia (AML) express Flt3 [39,40] and Flt3L has a strong stimulatory effect on these cells, enhancing colony growth when other cytokines are present at suboptimal levels [41]. Furthermore, around 35% of AML patients harbor a *FLT3* mutation [42,43], which often leads to constitutive activation of Flt3. In frame internal tandem duplications (ITD), in the juxta-membrane part of Flt3, account for 25–35% of the mutations in AML [44] and 5–10% of myelodysplastic syndrome (MDS) cases [45,46]. FLT3-ITD has also been associated with malignant transformation of MDS [45,47] and a poor prognostic outcome in AML [42,44,48,49,50], with the ratio of mutant to wild-type alleles having an impact [51]. The second most common *FLT3* mutations are missense point mutations in the tyrosine kinase domain which occur in approximately 5–10% of AML, 2–5% of MDS and 1–3% of acute lymphocytic leukemia (ALL) cases [46,51,52]. As to all of the above, selective Flt3 inhibitors are being examined as a means of treating some cases of AML [44,53].

Various populations of murine HSPC can be isolated by the use of cell surface markers, for example, the signaling lymphocytic activation molecule family of markers CD48, CD150 and CD224 [54]. In this study, we have used a combination of the quantitative reverse transcription polymerase chain reaction (qRT-PCR), to examine mRNA within single cells, and flow cytometry, for protein expression, to delineate the extent sub-populations of HSPC express Flt3. The expression of Flt3, or not, has been examined in relation to the expression of the receptors for erythropoietin (Epo) and M-CSF. The use of a triplex qRT-PCR assay and multi-color flow cytometry has revealed substantial heterogeneity of HSPC.

## 2. Results

### 2.1. Heterogeneous Expression of Flt3 Transcripts by Single HSPC

**Figure 1:**
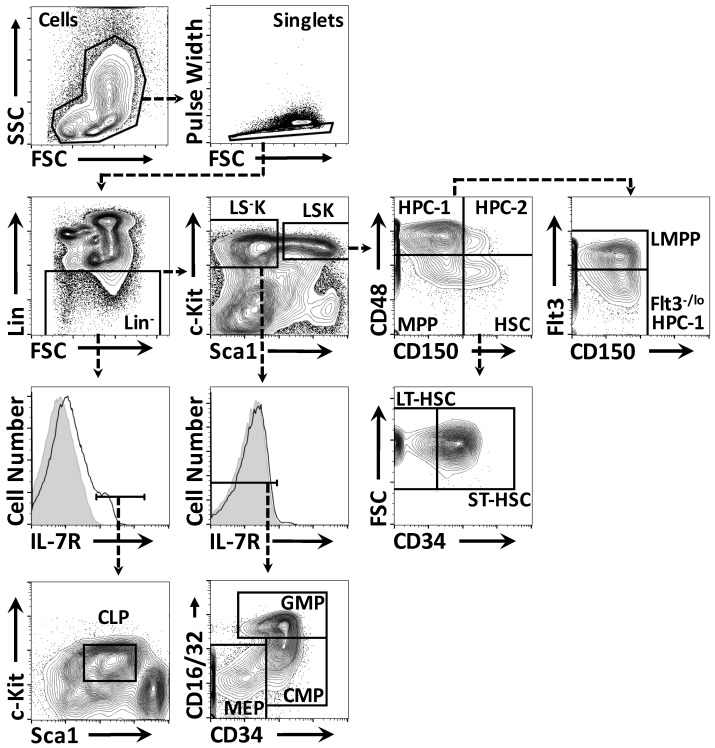
Isolation of hematopoietic stem and progenitor cells (HSPC) from mouse bone marrow. The gating strategies used to identify hematopoietic stem cells (HSC) and various progenitors are shown. The areas delineated by the black boxes in the scatter plots and solid black lines in the histograms indicate the population of cells gated. Dashed arrows point to further gating of these cells. Shaded areas in the histograms depict the isotype control staining. Lineage markers (Lin) included CD3ε, B220, CD11b, Ly-6G and TER-119. HSPC, hematopoietic stem and progenitor cells; LT-HSC, long-term reconstituting hematopoietic stem cell; ST-HSC, short-term reconstituting hematopoietic stem cell; HSC, hematopoietic stem cell; MPP, multipotent progenitor; HPC, hematopoietic progenitor cell; lymphoid-primed multipotent progenitors (LMPP), lymphoid-primed multipotent progenitor; common lymphoid progenitors (CLP), common lymphoid progenitor; common myeloid progenitors (CMP), common myeloid progenitor; GMP, granulocyte-macrophage progenitor; MEP, megakaryocyte-erythrocyte progenitor; LS^−^K, Lineage marker^−^ Sca1^−^ c-Kit^+^; LSK, Lineage marker^−^ Sca1^+^ c-Kit^+^; Flt3, fms-like tyrosine kinase 3; IL-7R, interleukin-7 receptor. SSC, side light scatter; FSC, forward light scatter.

Cell surface phenotypes that had been adapted from published profiles were used to identify and isolate the various populations of HSPC from murine bone marrow (Figure 1). The LSK compartment was divided into HSC (LSK CD150^+^ CD48^−^), MPP (LSK CD150^−^ CD48^−^), HPC-1 (LSK CD150^−^ CD48^+^) and HPC-2 (LSK CD150^+^ CD48^+^), as published by the Morrison group [54,55]. HSC were divided into LT-HSC (LSK CD150^+^ CD48^−^ CD34^−^) or ST-HSC (LSK CD150^+^ CD48^−^ CD34^+^) based on their expression of CD34 [1]. The gating strategy used to identify HPC-1 is similar to the one used by Adolfsson et al. to identify lymphoid-primed multipotent progenitors (LMPP), as these two cell populations express high levels of Flt3 at their surface [55,56]. Therefore, HPC-1 were divided into Flt3^−/lo^ HPC-1 (LSK CD150^−^ CD48^+^ Flt3^−/lo^) and Flt3^hi^ LMPP (LSK CD150^−^ CD48^+^ Flt3^hi^). The common myeloid progenitors (CMP; LS^−^K IL-7Rα^−^ CD16/32^lo^ CD34^hi^), granulocyte-macrophage progenitors (GMP; LS^−^K IL-7R^−^ CD16/32^hi^ CD34^hi^), MEP (LS^−^K IL-7Rα^−^ CD16/32^lo^ CD34^lo^) and common lymphoid progenitors (CLP; LS^int^K^int^ IL-7R^+^) were identified according to the strategies published by the Weissman group [57,58].

**Figure 2:**
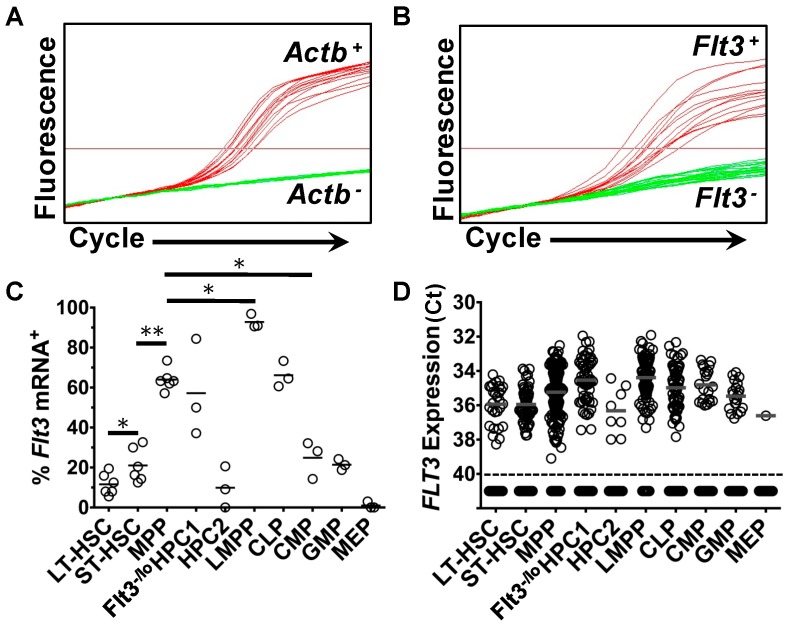
Expression of *Flt3* mRNA transcripts by HSPC. Analysis of *Flt3* mRNA expression by HSPC was carried out using duplex qRT-PCR assays specific for both *Actb* and *Flt3* transcripts. Reactions that did not give rise to a detectable amplification of *Actb* were removed from analysis. Representative amplification of: (**A**) the endogenous control mRNA, *Actb*; and (**B**) *Flt3* mRNA in single HSC are shown, where there is a clear distinction between cells that expressed the target gene (shown in red) and those that did not (shown in green); (**C**) The percentage of cells within each HSPC population that expressed *Flt3* mRNA; and (**D**) the levels of *Flt3* mRNA expressed by single HSPC. *Flt3* mRNA expression was estimated using the cycle at which the PCR signal crossed an arbitrary threshold (Ct) and each individual cell is represented by a single data point. Single cells that gave rise to a detectable amplification of *Actb* mRNA, but not *Flt3* mRNA, are plotted below the dotted lines. Data in (**C**,**D**) are the mean of the values obtained from *n =* 3–6 mice. In total, 248 LT-HSC, 252 ST-HSC, 297 MPP, 101 Flt3^−/lo^ HPC-1, 97 LMPP, 56 HPC-2, 94 CLP, 81 CMP, 98 GMP and 98 MEP were analyzed. *p* values were obtained by two-tailed non-parametric Student’s *t*-test, where * *p* < 0.05; ** *p* < 0.005,. HSC, hematopoietic stem cell; LT-HSC, long-term reconstituting hematopoietic stem cell; ST-HSC, short-term reconstituting hematopoietic stem cell; MPP, multipotent progenitor; LMPP, lymphoid-primed multipotent progenitor; CLP, common lymphoid progenitor; CMP, common myeloid progenitor; GMP, granulocyte-monocyte progenitor; MEP, megakaryocyte-erythrocyte progenitor; Flt3, fms-like tyrosine kinase 3; HSPC, hematopoietic stem and progenitor cell.

First, expression of *Flt3* mRNA by single cells within each of the above populations was investigated using qRT-PCR (Figure 2). Cells that expressed *Flt3* mRNA (*Flt3*mRNA^+^) and the endogenous control mRNA, *Actb*, could be readily identified (Figure 2A,B). In total, 1465 single cells were sorted and *Actb* mRNA was detected in 1416 samples (96.7%). Samples that did not contain detectable levels of *Actb* mRNA were removed from analysis.

Within the LT-HSC compartment, 11.6 ± 2.19% of cells (28 of 248 cells) expressed a detectable level of *Flt3* mRNA (Figure 2C,D). *Flt3* mRNA was expressed by 21 ± 3.4% (56 of 252 cells) of ST-HSC, which was significantly higher than the percentage of *Flt3*mRNA^+^ LT-HSC (*p* = 0.0476) (Figure 2C,D). Two hundred ninety-seven MPP were analyzed and 64 ± 2.2% (190 cells) of these cells were *Flt3*mRNA^+^ (Figure 2C,D). Analysis of progenitors that are associated with the myeloid pathways revealed a clear decrease in the percentage of *Flt3*mRNA^+^ cells as compared to the percentage of *Flt3*mRNA^+^ MPP (Figure 2C,D). Eighty-one CMP were analyzed and 21 of these cells expressed *Flt3* mRNA (24.9 ± 5.4%). Similarly, 21.4 ± 2.8% (21 of 98 cells) and 9.9 ± 6.0% (8 of 56 cells) of GMP and HPC-2 were *Flt3*mRNA^+^, respectively. Only one of the 98 MEP assayed (1 ± 1%) was found to be *Flt3*mRNA^+^. On the other hand, the fractions of *Flt3*mRNA^+^ cells within the lymphoid pathway-associated progenitor compartments were either comparable or greater than the percentage of *Flt3*mRNA^+^ cells within the MPP population (Figure 2C,D). Within the LMPP population, 92.8 ± 2.0 of cells (90 of 97 cells) were *Flt3*mRNA^+^, while 57.2 ± 14.1% (57 of 101 cells) and 66.2 ± 3.8% (62 of 94 cells) of Flt3^−/lo^ HPC-1 and CLP were *Flt3*mRNA^+^, respectively. Together, these data demonstrate that *Flt3* mRNA is expressed by cells within almost all HSPC populations, including HSC.

### 2.2. Expression of Flt3 Protein by HSPC Populations

**Figure 3:**
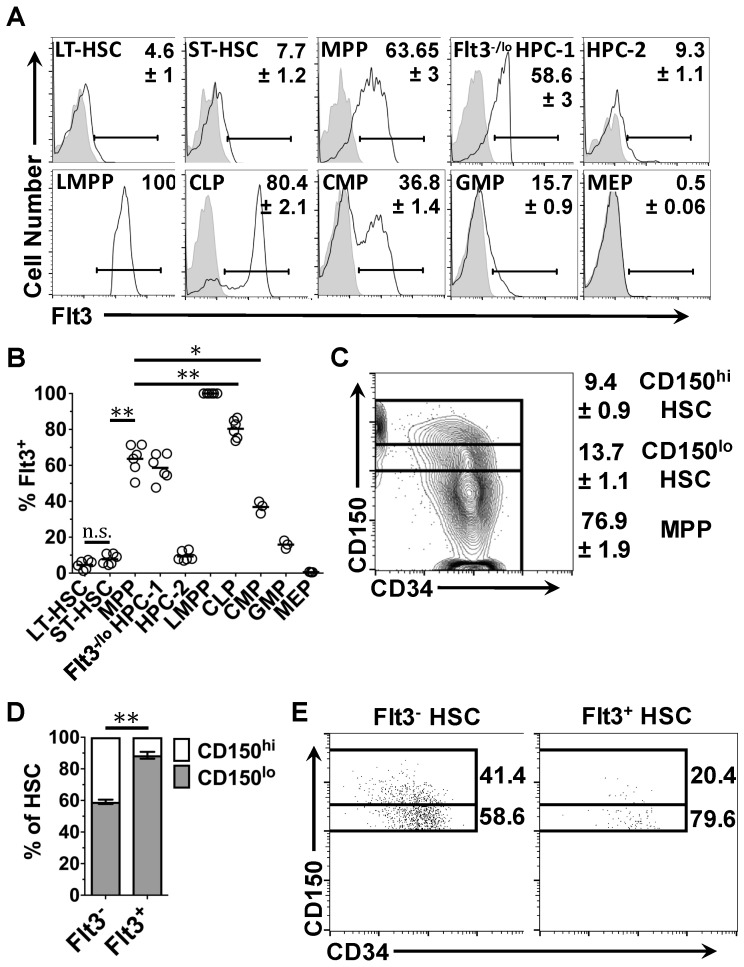
Cell surface expression of Flt3 protein by HSPC: (**A**) the gating strategy used to detect Flt3 on the surface of HSC and HPC populations (solid black line), compared to an isotype control (shaded histogram); (**B**) the percentage of cells within each HSPC compartment that expressed Flt3 protein at their surface; (**C**) the gating strategy used to identify LSK CD48^−^ cells with high (CD150^hi^) and low levels of CD150 (CD150^lo^) expressed at their surface and those lacking CD150 expression (MPP); (**D**) the percentages of Flt3^+^ and Flt3^−^ cells within the HSC compartment that express low and high levels of CD150 on their surface; and (**E**) the gating strategy in (**C**) applied to cells within the Flt3^+^ and Flt3^−^ HSC populations in a representative sample. Data in (**A**,**B**) and (**C**,**D**) are the mean of the values obtained from *n* = 3–6 and *n* = 6 mice, respectively. Values depicted in (**A**,**C**,**E**) represent the percentage of cells within each gate. Solid arrows in (**A**,**C**,**E**) indicate increasing cell number or the signal intensity in the designated channel. *p* values obtained by two-tailed non-parametric Student’s *t*-test, where ** *p* < 0.005. HSC, hematopoietic stem cell; LT-HSC, long-term reconstituting hematopoietic stem cell; ST-HSC, short-term reconstituting hematopoietic stem cell; MPP, multipotent progenitor; HPC, hematopoietic progenitor cell; LMPP, lymphoid-primed multipotent progenitor; CLP, common lymphoid progenitor; CMP, common myeloid progenitor; GMP, granulocyte-monocyte progenitor; MEP, megakaryocyte-erythrocyte progenitor; Flt3, fms-like tyrosine kinase 3; LSK, Lineage marker^−^ Sca1^+^ c-Kit^+^.

Next, the expression of Flt3 protein on the surface of bone marrow HSPC was investigated by flow cytometry (Figure 3). Flt3 protein was detected on the surface of 4.6 ± 1% of LT-HSC and 7.7 ± 1.2% of ST-HSC (Figure 3A,B). Interestingly, the expression of Flt3 protein by HSC correlated with the presence of a low level of CD150 at the cell surface (Figure 3D,E). A low level of expression of CD150 is associated with a reduced potential for self-renewal, and MegE development [59,60]. Within the MPP population, 63.7 ± 3% of cells had detectable levels of Flt3 at their cell surface (Figure 3A,B). In agreement with the gene expression analysis, the percentages of Flt3^+^ cells within the myeloid pathway-associated progenitor populations were less as compared to the MPP compartment (Figure 3A). Flt3 was detected on the surface of 36.8 ± 1.4% of CMP (Figure 3A,B). Within the HPC-2 and GMP populations, 9.3 ± 1.1% and 15.7 ± 0.9% of cells were Flt3^+^, respectively (Figure 3A). Flt3 was virtually absent from the MEP compartment (0.5 ± 0.06%) (Figure 3A). Lymphoid pathway-associated progenitor compartments contained either comparable or greater percentages of Flt3^+^ cells as compared to the MPP population. Flt3 was detected on the surface of 80 ± 2.1% of CLP, and 58.6 ± 3% of Flt3^−/lo^ HPC-1 (Figure 3A,B). As to their designated phenotype, 100% of LMPP were Flt3^+^ (Figure 3A). Similar to the *Flt3* mRNA expression data, these results show that Flt3 protein is expressed by all HSPC populations, apart from MEP, and is most commonly found on the surface of HPC with robust lymphoid potential.

### 2.3. Effects of Flt3 Stimulation on the Phosphorylation of Intracellular Ribosomal Protein S6 by HSC and MPP

**Figure 4:**
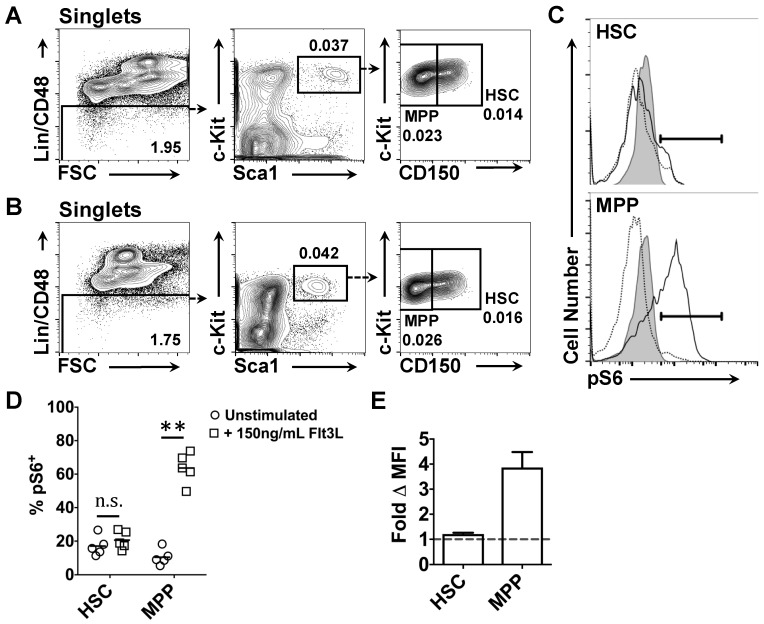
Changes in the phosphorylation of the ribosomal protein S6 in HSC and MPP following treatment with Flt3 ligand. Representative gating strategies used to analyze phosphorylated ribosomal protein S6 (pS6) showing: (**A**) unfixed; and (**B**) fixed and permeabilized samples. Gating of pS6^+^ HSC and MPP for treated (solid black line) and untreated cells (dotted black line) are shown in (**C**) and values for the percentages of positive cells are given in the text. Shaded histograms depict the isotype control; (**D**) The percentage of pS6^+^ cells in untreated and treated HSC and MPP; (**E**) The fold change in the MFI of pS6 staining in treated HSC and MPP compared to untreated cells. The areas delineated by solid black boxes in the scatter plots and solid black lines in the histograms indicate the population of cells gated. Dashed arrows point to further gating of these cells. Solid arrows in (**A**,**B**,**C**) indicate increasing cell number or signal intensity in the designated channel. Values depicted in (**A**,**B**) represent the percentage of total bone marrows cells within each gate. Data in (**D**,**E**) are the mean of the values obtained from *n* = 5 mice. *p* values obtained by two-tailed non-parametric Student’s *t*-test, where ** *p* < 0.005; n.s., non-significant. HSC, hematopoietic stem cell; MPP, multipotent progenitor; Flt3, fms-like tyrosine kinase 3; Flt3L, flt3 ligand; MFI, mean fluorescence intensity.

Murine HSC are thought not to express Flt3 and we identified a small fraction of cells within the LT-HSC and ST-HSC compartments that express both *Flt3* mRNA and Flt3 protein. To determine whether Flt3^+^ HSC and MPP respond to Flt3L in vitro, we adapted a previously described phospho-flow protocol for detecting phosphorylated S6 (pS6) in HSPC, as both the PI3K and RAS pathways converge to stimulate protein translation *via* phosphorylation of S6 [16,61,62,63]. Even though CD150 staining of whole bone marrow is compromised following cell fixation and permeabilization with acetone [63], CD150 staining was observed to be maintained within the LSK compartment when compared to untreated controls (Figure 4A,B). As such, we were able to monitor the levels of pS6 in the HSC and MPP populations.

After starvation in serum-free medium, HSC and MPP were stimulated in vitro with 150 ng/mL Flt3L for 7.5 min before being stained for pS6. For starved MPP stimulated with Flt3L, 63.7 ± 9.2% of cells expressed pS6 as compared to a value of 10.5 ± 1.38% for starved and untreated MPP (*p* = 0.0079) (Figure 4C,D). In contrast, Flt3L treatment resulted in only a marginal increase in the percentage of HSC expressing pS6 (from 17.1 ± 5.9% to 20.6 ± 5.4%, Figure 4C,D). In addition, the mean fluorescence intensity (MFI) of pS6 staining in starved and treated MPP was increased 3.9 ± 1.4-fold as compared to the pS6 MFI obtained for starved and untreated MPP, while the corresponding ΔMFI for HSC was 1.2 ± 0.1 (Figure 4E). These data demonstrate that Flt3L stimulates S6 phosphorylation in the majority of MPP. However, the effect of Flt3L on S6 phosphorylation in HSC is much less pronounced and is possibly due to the rarity of Flt3^+^ cells.

### 2.4. Flt3 Expression Identifies Distinct Sub-Populations of HSC and MPP

Other hematopoietic growth factor receptors, such as the receptors for Epo (EpoR) and M-CSF (M-CSFR), have been implicated in the pathogenesis of leukemia [64,65,66]. Both EpoR and M-CSFR are known to be expressed by HSC, and there is evidence to support their role in the development of HSC [67,68]. Therefore, we examined whether Flt3 and EpoR or Flt3 and M-CSFR are co-expressed by cells within the HSC compartment, or if the expression of these receptors identifies distinct sub-populations of HSC.

**Figure 5:**
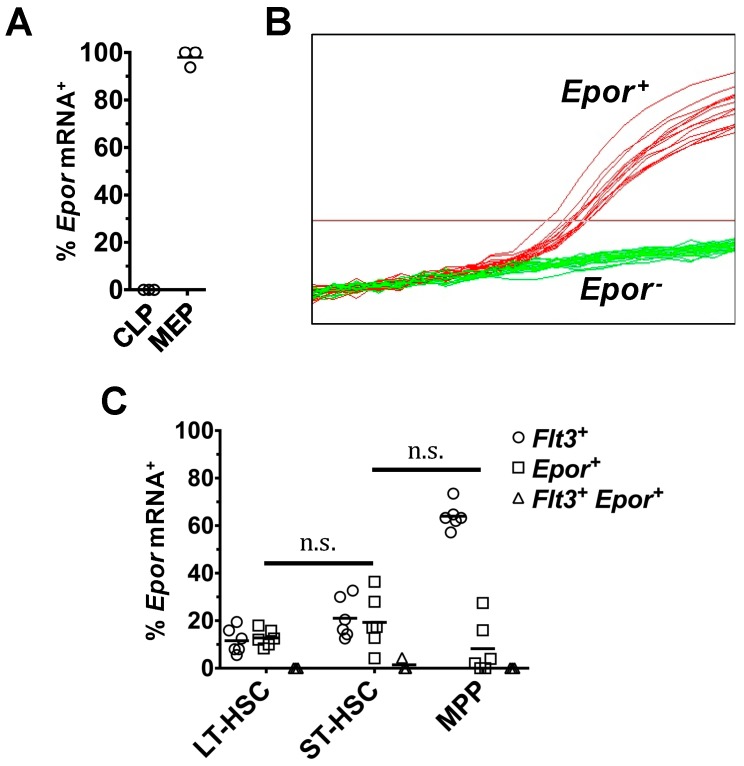
*Flt3* and *Epor* transcripts are rarely co-expressed by LT-HSC, ST-HSC and MPP. Analysis of *Flt3* and *Epor* co-expression by LT-HSC, ST-HSC and MPP was carried out using a triplex qRT-PCR assay specific for *Actb*, *Flt3*, and *Epor* mRNA. Reactions that did not give rise to a detectable amplification of *Actb* were removed from analysis. (**A**) The percentage of CLP and MEP that expressed *Epor* mRNA; (**B**) the amplification of *Epor* mRNA in single ST-HSC where there is a clear distinction between cells that expressed the *Epor* (shown in red) and those that did not (shown in green); and (**C**) the percentage of LT-HSC, ST-HSC and MPP that co-expressed both *Flt3* and *Epor* mRNA. The total fraction of *Flt3*mRNA^+^ and *Epor*mRNA^+^ are also shown. Data in (**A**) are the mean of the values obtained from *n* = 3 mice. Data in (**C**) are the mean of the values obtained from *n* = 3–6 mice. *p* values obtained by two-tailed non-parametric Student’s *t*-test, where n.s., non-significant. LT-HSC, long-term reconstituting hematopoietic stem cell; ST-HSC, short-term reconstituting hematopoietic stem cell; MPP, multipotent progenitor; MEP, megakaryocyte-erythrocyte progenitor; CLP, common lymphoid progenitor; Flt3, fms-like tyrosine kinase 3; Epor, erythropoietin receptor.

To determine if HSC and MPP co-express the genes encoding Flt3 and EpoR, we designed a triplex qRT-PCR assay to measure the levels of *Actb*, *Flt3* and *Epor* mRNAs in single cells. The *Epor* qRT-PCR assay detected *Epor* mRNA expression in 0 of 94 CLP and 96 of 98 MEP, suggesting a high degree of specificity of the assay (Figure 5A). As with cells expressing *Actb* and *Flt3* mRNA, cells expressing *Epor* mRNA (*Epor*mRNA^+^) were readily identified in the HSC and MPP compartments (Figure 5B). One hundred thirty-six LT-HSC, 139 ST-HSC and 148 MPP were analyzed for expression of *Flt3* mRNA and *Epor* mRNA (Figure 5C). For LT-HSC, 12.8 ± 1.46% of cells were *Epor*mRNA^+^ and none of these cells co-expressed both *Flt3* and *Epor* mRNA. Of the ST-HSC analyzed, 19.3 ± 4.6% of cells expressed *Epor* mRNA. Notably, 2 of the 139 ST-HSC (1.5 ± 2.5%) analyzed for *Flt3* and *Epor* mRNA co-expressed both mRNAs. Within the MPP compartment, 8.2 ± 4.6% were *Epor*mRNA^+^, and none of them co-expressed *Flt3* mRNA. We could not examine the co-expression of Flt3 and EpoR protein at the cell surface because of the lack of an antibody to EpoR.

**Figure 6:**
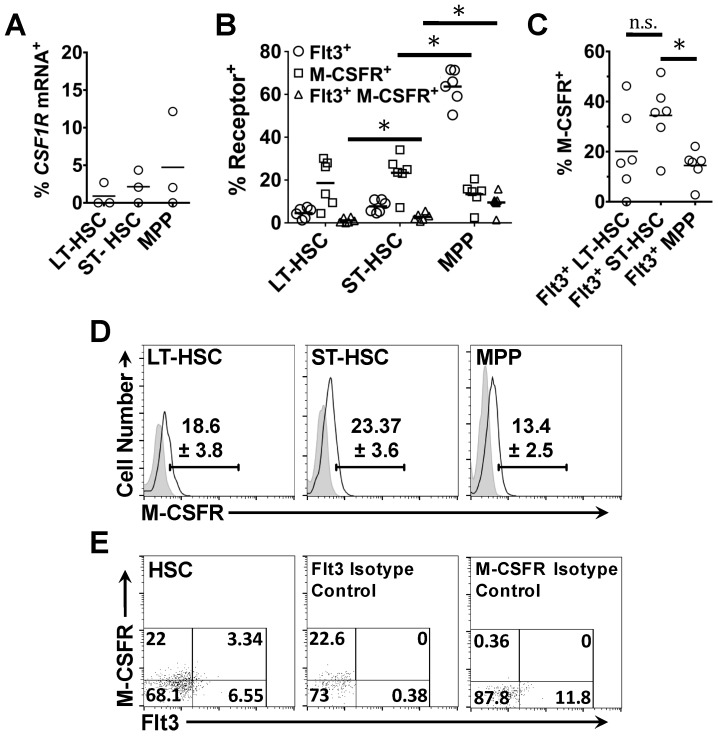
Flt3 and M-CSFR protein are primarily expressed by distinct sub-populations of LT-HSC and ST-HSC: (**A**) analysis of *Csf1r* mRNA expression by single LT-HSC, ST-HSC and MPP. Data are the mean of the values obtained from *n* = 3 mice; (**B**) the percentage LT-HSC, ST-HSC and MPP that expressed Flt3, M-CSFR or both of these receptors at their surface; (**C**) the percentage of Flt3^+^ LT-HSC, Flt3^+^ ST-HSC and Flt3^+^ MPP that expressed M-CSFR at their surface; and (**D**) the gating strategy used to identify M-CSFR^+^ LT-HSC, M-CSFR^+^ ST-HSC, and M-CSFR^+^ MPP (solid black line). Shaded histograms depict the isotype control. Scatter plots in (**E**) represent the gating strategy used to identify HSC that co-expressed Flt3 and M-CSFR at their surface. Isotype controls for both Flt3 and M-CSF staining within the HSC compartment are also shown. Solid arrows in (**D**,**E**) indicate increasing cell number or signal intensity in the designated channel. Data in (**B**–**D**) are the mean of the values obtained from *n* = 6 mice. Values in (**D**,**E**) are the percentages of cells within the corresponding gates. *p* values were obtained by two-tailed non-parametric Student’s *t*-test, where * *p* < 0.05; n.s., non-significant. HSC, hematopoietic stem cell; LT-HSC, long-term reconstituting hematopoietic stem cell; ST-HSC, short-term reconstituting hematopoietic stem cell; MPP, multipotent progenitor; Flt3, fms-like tyrosine kinase 3; M-CSFR, macrophage colony stimulating factor receptor.

We then investigated whether HSC and MPP co-expressed the genes encoding Flt3 and M-CSFR (*Csf1r*) and detected very few cells that expressed *Csf1r* mRNA (*Csf1r*mRNA^+^) in these compartments. *Csf1r* mRNA was detected in 0.9 ± 0.9% (1 of 122 cells) of LT-HSC, 2.14 ± 1.3% (2 of 118 cells) of ST-HSC, and 4.8 ± 3.8% (7 of 145 cells) of MPP (Figure 6A). As to the few *Csf1r*mRNA^+^ cells, either the *Csf1r* qRT-PCR assays was not sensitive enough to detect *Csf1r* mRNA expression in HSC and MPP or these cells rarely transcribe the *Csf1r* gene. Mossadegh-Keller et al. have also investigated whether HSC express *Csf1r* mRNA and have reported that very few cells are *Csf1r*mRNA^+^, suggesting the latter [68]. Surprisingly, a significant proportion of HSC and MPP expressed M-CSFR protein (Figure 6B,D). We therefore examined whether cells within the HSC and MPP compartments co-expressed Flt3 and M-CSFR protein at their surface.

Within the LT-HSC population, 18.6 ± 3.8% of cells were M-CSFR^+^ and only 1.12 ± 0.48% of these cells expressed both Flt3 and M-CSFR. M-CSFR was detected at the surface of 23.4 ± 3.6% of ST-HSC, and 2.8 ± 0.7% co-expressed both Flt3 and M-CSFR protein (Figure 6B). Of the MPP analyzed, 13.4 ± 2.46% of cells expressed M-CSFR at their surface which was significantly less than the proportion of M-CSFR^+^ cells in the ST-HSC population (*p* = 0.0313), though the fraction of Flt3^+^ M-CSFR^+^ MPP (9.9 ± 1.9%) was greater than when compared to the ST-HSC population (*p* = 0.0313) (Figure 6B). As Flt3 is only expressed by a small percentage of HSC, we also examined Flt3 and M-CSFR co-expression as a percentage of Flt3^+^ cells within the HSC and MPP compartments (Figure 6C). Of the Flt3^+^ LT-HSC and Flt3^+^ ST-HSC, 20.1 ± 6.87% and 34.47 ± 5.35% of cells also expressed M-CSFR, respectively. Of the Flt3^+^ MPP population, 14.46 ± 2.62% also expressed M-CSFR at their surface. These data show that Flt3, EpoR and M-CSFR are selectively expressed during early hematopoiesis and that expression of these receptors identifies novel subpopulations of early HSPC.

## 3. Discussion

In this study, the expression of *Flt3* mRNA and Flt3 protein by the various populations of HSPC from the murine bone marrow has been investigated. HSC and MPP were then analyzed to determine whether Flt3 and EpoR or Flt3 and M-CSFR are co-expressed by cells within these compartments. The findings demonstrate a large degree of heterogeneity within the HSPC compartments, and distinct sub-populations of HSC and MPP can be identified by their expression of Flt3, EpoR and M-CSFR.

**Figure 7:**
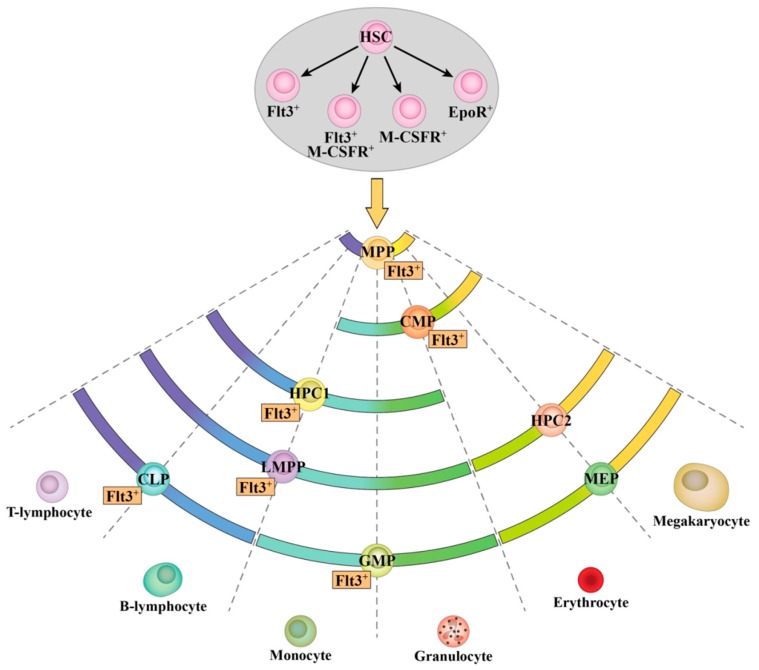
Heterogeneous expression of lineage affiliated receptors Flt3, EpoR and M-CSFR in murine HSPC. Four populations of HSC were identified in this study based on the expression of Flt3, EpoR and M-CSFR (as indicated by the black arrows). These populations are depicted within the shaded area. MPP and HPC are downstream of HSC (as indicated by the yellow arrow) and are heterogeneous as to their expression of Flt3, and this is illustrated using the pairwise model (described in [72] and [71]). The maturation potential of HSPC is depicted by the colored arcs. Expression of Flt3 protein and *Flt3* mRNA was strongly associated with lymphoid-GM potential, as expected. Flt3 expression was rarely observed within the HPC-2 compartment, and was virtually absent from MEP. In this study, HPC-1 and LMPP were defined as LSK CD48^+^ CD150^−^ and LSK CD48^+^ CD150^−^ Flt3^hi^, respectively. The percentages of *Flt3mRNA*^+^/Flt3^+^ cells within each compartment are as follows: LT-HSC, 11.6 ± 2.2%/4.6 ± 1%; ST-HSC, 21 ± 3.4%/7.7 ± 1.2%; MPP, 64 ± 2.2%/63.7 ± 3%; Flt3^−/lo^ HPC-1, 57.2 ± 14.1%/58.6 ± 3%; HPC-2, 9.9 ± 6.0%/9.3 ± 1.1%; LMPP, 92.8 ± 2.0%/100%; CLP, 66.2 ± 3.8%/80 ± 2.1%; CMP, 24.9 ± 5.4%/36.8 ± 1.4%; GMP, 21.4 ± 2.8%/15.7 ± 0.9%; MEP, 1 ± 1%/0.5 ± 0.06%. HSC, hematopoietic stem cell; LT-HSC, long-term reconstituting hematopoietic stem cell; ST-HSC, short-term reconstituting hematopoietic stem cell; HPC, hematopoietic progenitor cell; MPP, multipotent progenitor; LMPP, lymphoid-primed multipotent progenitor; CLP, common lymphoid progenitor; CMP, common myeloid progenitor; GMP, granulocyte-macrophage progenitor; MEP, megakaryocyte-erythrocyte progenitor; Flt3, fms-like tyrosine kinase 3; M-CSFR, macrophage colony-stimulating factor receptor; EpoR, erythropoietin receptor; LSK, Lineage marker^−^ Sca1^+^ c-Kit^+^.

Flt3 is generally considered to be absent from the surface of HSC in the adult mouse [5,6,69], though recent studies have identified *Flt3* expression within the LSK CD150^+^ CD48^−^ stem cell compartment [9,70]. Furthermore, Chu et al. have shown that expression of FLT3-ITD under the control of the endogenous Flt3 promoter results in the depletion of the murine stem cell pool [70]. Here, the HSC compartment was divided into LT-HSC (LSK CD150^+^ CD48^−^ CD34^−^) and ST-HSC (LSK CD150^+^ CD48^−^ CD34^+^) and both of these populations contain a small percentage of cells that express *Flt3* mRNA and Flt3 protein. Downstream of HSC, Flt3 expression delineates murine lymphoid/myeloid progenitors from those progressing along the MegE pathway; this is illustrated by Figure 7, which maps HSPC populations and Flt3 expression to the pairwise model (described in [71] and [72]). In humans, Flt3 has been reported to be expressed by HSC [73]. Taking these findings together, the leukemias with Flt3-activating mutations, which include AML and ALL, might well have arisen from the Flt3^+^ primitive stem cell compartment of the bone marrow. Therefore, the populations identified here are important to understanding the origin of leukemia and their potential lineage affiliation is pertinent to disease progression. This viewpoint is also important to studies that make use of FLT3-ITD mouse models.

Previous characterization of Flt3^+^ cells within the primitive cell compartments of the adult murine bone marrow has indicated that these cells have limited self-renewal capacity [5,7,9]. Indeed, Flt3 expression within the HSC compartment correlates with a low level of CD150 at the cell surface (Figure 3D,E) which is associated with a reduced self-renewal ability [59,60]. However, the reconstitution assays performed in the above studies are limited by their use of bulk Flt3^+^ LSK cells, which contain very few cells that are Flt3^+^ LSK CD150^+^ CD48^−^, and their assessment of self-renewal capacity by just monitoring myeloid reconstitution. Using their FlkSwitch model, the Forsberg group have recently identified a transient population of fetal Flt3^+^ HSC that preferentially give rise to lymphoid cells [74]. Like the Flt3^+^ cells in the adult murine HSC compartment identified here, fetal Flt3^+^ HSC express a low level of CD150 at their surface. Hence, Flt3 expression in the adult HSC compartment may identify a rare population of lymphoid-biased HSC. Fetal Flt3^+^ HSC do not persist in the bone marrow past 8 weeks of age, so it is unlikely that the phenotypic Flt3^+^ HSC identified here are fetal Flt3^+^ HSC that have persisted into late adulthood [74].

Investigation of the expression of *Epor* mRNA and M-CSFR protein identified subpopulations of cells within the LT-HSC, ST-HSC and MPP compartments (Figure 7). Co-expression of *Flt3* mRNA and *EpoR* mRNA was exceedingly rare, indicating that Flt3 and EpoR expression almost exclusively identify distinct subpopulations of multipotent progenitors. Co-expression of M-CSFR and Flt3 by early HSPC was more common, which was expected since both Flt3 and M-CSFR are associated with the development of myelomonocytic progenitors. Conventionally, HSC are thought to gradually commit to mature cell fates by transitioning through various intermediate progenitor states. However, long-term reconstituting lineage-biased and lineage-restricted progenitors have been identified in the HSC compartment of the mouse bone marrow [59,60,75,76,77,78,79,80,81,82,83], and megakaryocyte specification occurs primarily within the HSC compartment in humans [84]. These findings suggest that lineage specification occurs at a very early stage of hematopoiesis, without HSC having to transit through a succession of intermediate progenitors. Indeed, a recent study of human HSPC supports this viewpoint [85]. The expression of lineage-associated growth factor receptors by HSC might represent adoption of a fate, providing a selective advantage to cells as to the presence of a particular growth factor. Some hematopoietic growth factor receptors, including Flt3 and M-CSFR, have been used to identify lineage-biased subpopulations within currently defined HPC [83,86,87,88,89], and therefore, as already stated in regard to Flt3 expression, EpoR and M-CSFR might be used to enrich for functionally distinct HSC.

As has been previously reported, Flt3 expression dramatically increases during the HSC-to-MPP transition. Approximately 60% of MPP (LSK CD150^−^ CD48^−^) express Flt3 and phosphorylate S6 following stimulation with Flt3L [55]. Progenitors downstream of HSC and MPP, other than the MEP, were also found to be heterogeneous as to their expression of Flt3 (Figure 7); the patterns reported here are largely similar to previously published data [55,56,87,90]. As mentioned above, growth factor receptor expression has been used to identify lineage-biased subpopulations of HPC. Further study of the populations identified here might be useful in resolving whether progenitor populations that are viewed as having a number of different lineage options are a uniform population of cells or a mixture of cells with individual lineage potentials.

As expected, Flt3 expression is linked to populations that have robust lymphoid, granulocyte and myelomonocytic potential. Conversely, the frequency of Flt3 expression decreases as cells differentiate towards a MegE fate. This is typified by HPC-1 (LSK CD150^−^ CD48^+^), which includes the Flt3^−/lo^ HPC-1 and LMPP populations analyzed in this study, and HPC-2 (LSK CD150^+^ CD48^+^). The HPC-1 compartment contains progenitors that preferentially give rise to lymphoid cells and possess limited MegE potential, and Flt3 expression was most commonly detected within this compartment [55]. HPC-2 have significant myeloid potential and a reduced lymphoid potential when compared to HPC-1, and Flt3 expression was infrequently detected in the HPC-2 compartment [55]. The importance of Flt3 expression by lymphoid and GM lineages, as opposed to MegE lineage, is emphasized by the findings from Flt3L-Tg mice [38].

The findings described here, and in a number of other studies, demonstrate the heterogeneity of HSPC. Similarly, compartments that were once thought to comprise of a uniform population of cells have been shown to contain a mixture of cells with varying lineage potentials and cell-intrinsic states [84,85,86,91]. Moreover, HSC at different stages of the cell cycle display functional heterogeneity [92,93], and hematopoietic progenitors fluctuate between states with differing capacities for particular cell fates [94], highlighting the dynamic nature of cells. These matters need to be considered when using techniques that are reliant on cell surface phenotypes which only provide information about a cell at a single moment in time. In this regard, single cell fate mapping and imaging techniques will be integral to the functional characterization of distinct HSPC subpopulations, such as those identified in this study. Such work is of importance for our understanding of malignant transformation in hematopoiesis and the heterogeneity of leukemia.

## 4. Materials and Methods

### 4.1. Animals

Male C57/BL6 mice were purchased from Harlan Laboratories, UK, and housed in the Biomedical Services Unit at The University of Birmingham, UK. All mice were treated in accordance with Home Office guidelines, and were culled by cervical dislocation between 8–14 weeks of age. The use of bone marrow cells from normal mice was conducted under a licence provided to Ciaran Mooney (No. 34942, 18 October 2013) having completed a programme of training approved by the Universities Accreditation Scheme.

### 4.2. Flow Cytometry

Bone marrow cells were flushed from mouse tibias, femurs and humeri using flow cytometry buffer (Dulbecco’s phosphate buffered (DPBS) without Ca^+^/Mg^+^ (Thermo Fisher Scientific, Waltham, MA, USA) which contained 2% fetal calf serum (FCS) (Thermo Fisher Scientific) and 2 mM ethylenediaminetetraacetic acid (Sigma Aldrich, St. Louis, MO, USA)). Bone marrow cells were strained using 70 μM Cell strainers (BD biosciences, San Diego, CA, USA) and erythrocytes were lysed using ACK Lysing Buffer (Thermo Fisher Scientific). Cells were then stained with fluorescently-labeled antibodies in flow cytometry buffer for 1 h on ice. Other than when CD16/32 expression was examined using fluorescently-labeled antibodies, cells were incubated with purified anti-CD16/CD32 (clone 93, BioLegend, San Diego, CA, USA) for 20 min to block CD16 (FcγIII) and CD32 (FcγII) receptors prior to staining. The following antibodies were used to analyze the phenotype of cells; anti-CD3ε-AF488 (clone 17A2, BioLegend), anti-B220-AF488 (clone RA3-6B2, BioLegend), anti-CD11b-AF488 (clone M1/70, BioLegend), anti-TER-119-AF488 (clone TER-119, BioLegend), anti-Ly-6G-AF488 (clone RB6-8C5, BioLegend), anti-CD16/32-PE (clone 93, BioLegend), anti-CD34-APC (clone RAM34, eBiosciences, San Diego, CA USA), anti-CD48-AF488 (clone HM48-1, BioLegend), anti-CD48-APC-Cy7 (clone HM48-1, BioLegend), anti-CD115(M-CSFR)-BV421 (cloneAFS98, BD Biosciences), anti-CD117-PE-CF594 (clone 2B8, BD Biosciences), anti-CD127-PE-Cy7 (clone A7R34, eBiosciences), anti-CD135-PE (clone A2F10, eBiosciences), anti-CD150-PE (clone TC15-12F12.2, BioLegend), anti-CD150-Pacific Blue (clone TC15-12F12.2, BioLegend), anti-Sca1-PE-Cy7 (clone D7, eBiosciences), anti-pS6(Ser235/Ser236)-PE (clone D57.2.2E, Cell Signalling). Data were acquired using a CyAN FACS Analyser (Beckman Coulter, Fullerton, CA, USA) controlled by Summit v4.3 software.

### 4.3. Fluorescence Activated Cell Sorting (FACS)

Prior to sorting, stained bone marrow cells were suspended in flow cytometry buffer containing 10% FCS, filtered using Partec CellTrics sterile filters (Sysmex-Partec, Görlitz, Germany) and stored on ice. All of the HSPC populations were sorted twice to ensure high purity. Cells were sorted into LightCycler 480 384-well plates (Roche, Basel, Switzerland) for qRT-PCR analysis or into culture medium for in vitro manipulation. Sorting was carried out using either a MoFlo High Speed Sorter (Beckman Coulter) controlled by Summit v4.3 software or a MoFlo Astrios (Beckman Coulter) controlled by Summit v6.2.3 software.

### 4.4. Single Cell Quantitative Reverse Transcription Polymerase Chain Reaction

Single cells were sorted into the wells of LightCycler 480 384-well plates (Roche) containing 1μL of UltraPure DNase/RNase-Free Distilled Water (Thermo Fisher Scientific) for lysis. Gene expression analysis was carried out using a one-step QuantiTect Multiplex RT-PCR Kit (Qiagen, Hilden, Germany) containing limiting concentrations of primers and hydrolysis probes specific for the *Actb* and *Flt3* transcripts. qRT-PCR reactions were carried out and analyzed on a LC480 II instrument (Roche). The program used for qRT-PCR was as follows: reverse transcription, 50 °C for 20 min; polymerase activation, 95 °C for 15 min; and 40–50 cycling steps of denaturation at 95 °C for 45 s, and annealing/extension at 60 °C for 45 s. Oligonucleotides used for qRT-PCR analysis were purchased from Biosearch Technologies (Novato, California, USA) and were as follows; *Actb* forward primer, 5′-CAGCTTCTTTGCAGCTCCTTC-3′; *Actb* reverse primer, 5′-CGACCAGCGCAGCGATAT-3′; *Actb* hydrolysis probe, 5′-CACCAGTTCGCCATGGA-3′; *Flt3* forward primer, 5′-ATCAGCGGGAAAGCCATCATC-3′; *Flt3* reverse primer, 5′-GGGCACACTGGAGGTCTTCT-3′; *Flt3* hydrolysis probe, 5′-TCCTCGCACCATTCGGTA-3′; *Epor* forward primer, 5′- GCAGGAGGGACACAAAGGGT-3′; *Epor* reverse primer, 5′- GGGCTCAGACCAGGCACT-3′; *Epor* hydrolysis probe, 5′-CTCGAACAGCGAAGGTGTAGCGC-3′; *Csf1r* forward primer, 5′-ACCTGTCCTGGTCATCACT-3′; *Csf1r* reverse primer, 5′-AACCTCTTGGGAGCCTGTACTCAC-3′; *Csf1r* hydrolysis probe, 5′-GCATAGCCTCGGCCTTCCTT-3′.

### 4.5. Phospho-Flow Analysis

Bone marrow cells were stained to identify LT-HSC, ST-HSC and MPP and then washed using serum-free DPBS (without Ca^+^/Mg^+^) three times before being starved in serum-free Iscove’s Modified Dulbecco’s Medium (Thermo Fisher Scientific) for 3 h. After the starvation period, each sample was divided in half and one half was stimulated with 150 ng/mL Flt3L for 7.5 min. All samples were then promptly fixed in 1.6% EM-grade paraformaldehyde (PFA) (VWR International, Radnor, PA, USA) for 15 min. Cells were then permeabilized using ice-cold acetone for 15 min at −20 °C, and then stained using an anti-pS6(Ser235/Ser236)-PE (clone D57.2.2E, Cell Signaling Technology, Danvers, MA, USA) and in DPBS containing 2% FCS for 1 h on ice. Cells were washed twice with DPBS containing 2% FCS before data acquisition on a CyAN FACS Analyser (Beckman Coulter) controlled by Summit v4.3 software. During analysis, pS6 staining of stimulated and unstimulated samples of cells from a single mouse was compared.

## 5. Conclusions

Analyses of single cells within the HSC and HPC bone marrow compartment of C57/BL6 mice, by multiplex RT-PCR for mRNA and flow cytometry for surface protein, has revealed that these cells are heterogeneous in regard to expression of Flt3. Analysis of M-CSFR and EpoR expression within the multipotent compartments of the bone marrow cells revealed further heterogeneity. Flt3 and EpoR are rarely co-expressed whereas co-expression of Flt3 and M-CSFR occurs more commonly. The expression of these receptors for lineage-associated growth factors by HSC indicates their affiliation to or adoption of a fate. The distinct subpopulations of HSPC identified are important to understanding the disease origin and heterogeneity of leukemia.
